# Supplementary material for: Different Strategies of Preimplantation Genetic Testing for Aneuploidies in Women of Advanced Maternal Age: A Systematic Review and Meta-Analysis
Source: J Clin Med. 2021 Aug 30;10(17):3895. doi: 10.3390/jcm10173895 (PMC8432243; doi:10.3390/jcm10173895)

Supplemental Figure S1. Risk of bias summary.

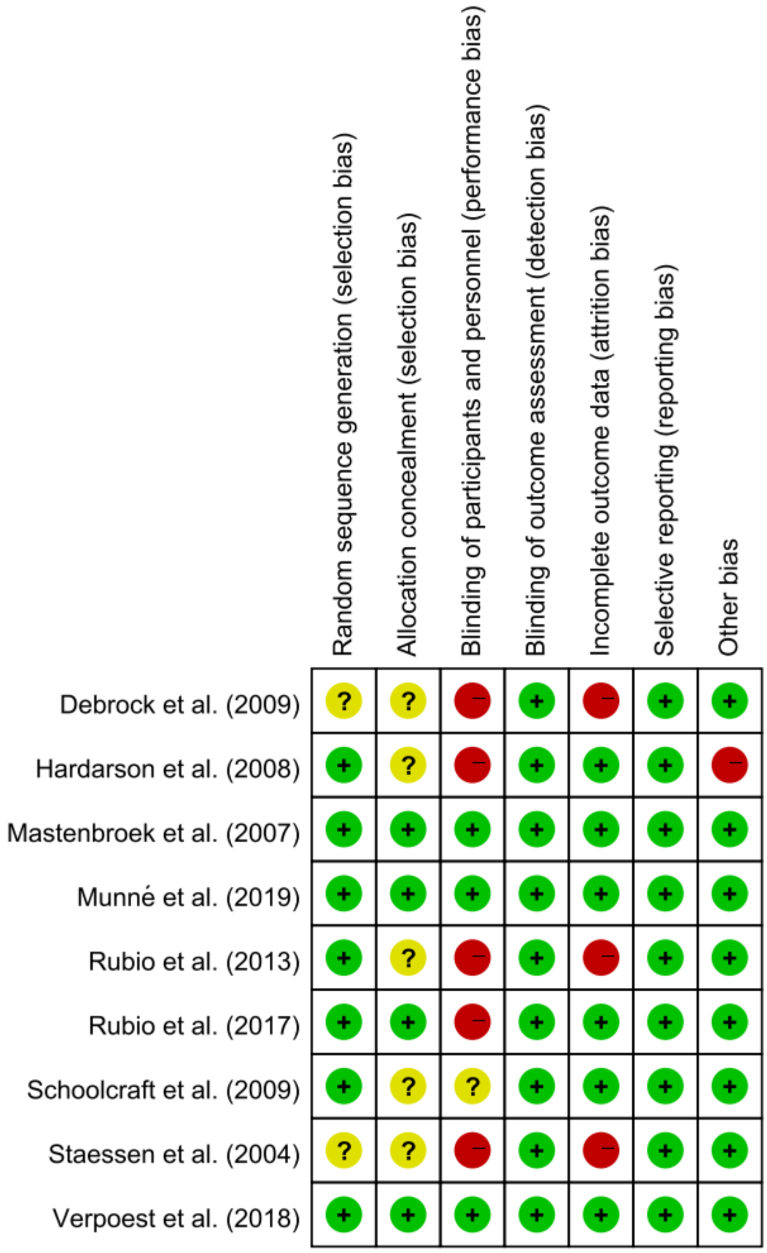

Supplemental Figure S2. The effect of PGT-A on ongoing pregnancy rate. (**a**) PGT-A with different techniques of genetic testing. (**b**) PGT-A with different stages of embryo biopsy.

a

Study or Subgroup	PGT-A Events	PGT-A Total	Control Events	Control Total	Weight	Risk Ratio M-H, Random, 95% CI	Year
**FISH**							
Staessen et al. (2004)	22	199	29	190	11.1%	0.72 [0.43, 1.21]	2004
Mastenbroek et al. (2007)	52	206	74	202	14.9%	0.69 [0.51, 0.93]	2007
Hardarson et al. (2008)	3	56	10	53	4.0%	0.28 [0.08, 0.98]	2008
Debrock et al. (2009)	6	44	10	50	6.0%	0.68 [0.27, 1.72]	2009
Schoolcraft et al. (2009)	16	32	16	30	11.6%	0.94 [0.58, 1.52]	2009
Rubio et al. (2013)	31	93	17	90	11.1%	1.76 [1.05, 2.96]	2013
**Subtotal (95% CI)**		**630**		**615**	**58.6%**	**0.83 [0.57, 1.20]**	
Total events	130		156				
Heterogeneity: Tau² = 0.12; Chi² = 13.33, df = 5 (P = 0.02); I² = 62%							
Test for overall effect: Z = 0.98 (P = 0.32)							
**Comprehensive chromosomal screening**							
Rubio et al. (2017)	36	100	23	105	12.3%	1.64 [1.05, 2.57]	2017
Verpoest et al. (2018)	50	205	45	191	13.9%	1.04 [0.73, 1.47]	2018
Munné et al. (2019)	62	122	54	145	15.2%	1.36 [1.04, 1.79]	2019
**Subtotal (95% CI)**		**427**		**441**	**41.4%**	**1.30 [1.03, 1.65]**	
Total events	148		122				
Heterogeneity: Tau² = 0.01; Chi² = 2.81, df = 2 (P = 0.25); I² = 29%							
Test for overall effect: Z = 2.18 (P = 0.03)							
**Total (95% CI)**		**1057**		**1056**	**100.0%**	**1.00 [0.76, 1.33]**	
Total events	278		278				
Heterogeneity: Tau² = 0.11; Chi² = 26.47, df = 8 (P = 0.0009); I² = 70%							
Test for overall effect: Z = 0.03 (P = 0.97)							
Test for subgroup differences: Chi² = 4.00, df = 1 (P = 0.05), I² = 75.0%							

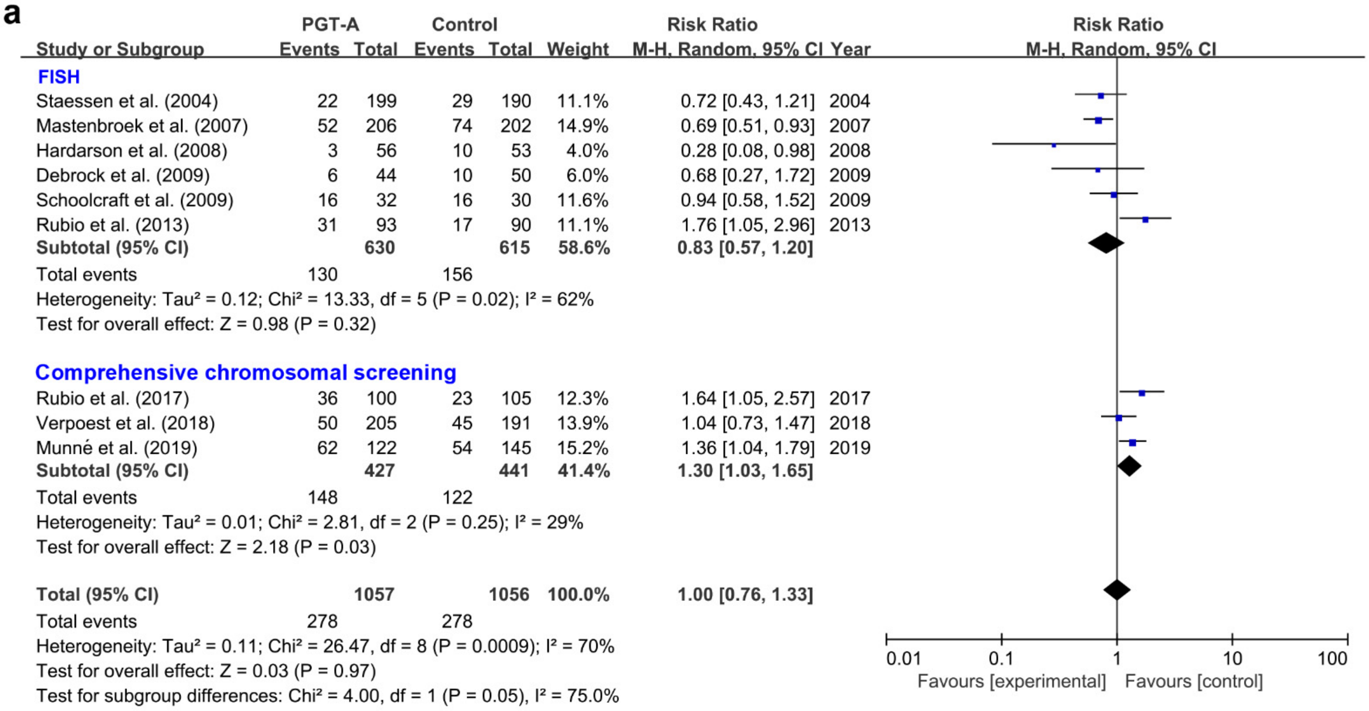

b

Study or Subgroup	PGT-A Events	PGT-A Total	Control Events	Control Total	Weight	Risk Ratio M-H, Fixed, 95% CI	Year
**Polar body biopsy**							
Verpoest et al. (2018)	50	205	45	191	16.9%	1.04 [0.73, 1.47]	2018
**Subtotal (95% CI)**		**205**		**191**	**16.9%**	**1.04 [0.73, 1.47]**	
Total events	50		45				
Heterogeneity: Not applicable							
Test for overall effect: Z = 0.19 (P = 0.85)							
**Cleavage stage biopsy**							
Staessen et al. (2004)	22	199	29	190	10.7%	0.72 [0.43, 1.21]	2004
Mastenbroek et al. (2007)	52	206	74	202	27.1%	0.69 [0.51, 0.93]	2007
Hardarson et al. (2008)	3	56	10	53	3.7%	0.28 [0.08, 0.98]	2008
Schoolcraft et al. (2009)	16	32	16	30	6.0%	0.94 [0.58, 1.52]	2009
Debrock et al. (2009)	6	44	10	50	3.4%	0.68 [0.27, 1.72]	2009
Rubio et al. (2013)	31	93	17	90	6.3%	1.76 [1.05, 2.96]	2013
Rubio et al. (2017)	36	100	23	105	8.1%	1.64 [1.05, 2.57]	2017
**Subtotal (95% CI)**		**730**		**720**	**65.3%**	**0.92 [0.76, 1.10]**	
Total events	166		179				
Heterogeneity: Chi² = 21.00, df = 6 (P = 0.002); I² = 71%							
Test for overall effect: Z = 0.95 (P = 0.34)							
**Blastocyte stage biopsy**							
Munné et al. (2019)	62	122	54	145	17.9%	1.36 [1.04, 1.79]	2019
**Subtotal (95% CI)**		**122**		**145**	**17.9%**	**1.36 [1.04, 1.79]**	
Total events	62		54				
Heterogeneity: Not applicable							
Test for overall effect: Z = 2.22 (P = 0.03)							
**Total (95% CI)**		**1057**		**1056**	**100.0%**	**1.02 [0.88, 1.17]**	
Total events	278		278				
Heterogeneity: Chi² = 26.47, df = 8 (P = 0.0009); I² = 70%							
Test for overall effect: Z = 0.23 (P = 0.82)							
Test for subgroup differences: Chi² = 5.66, df = 2 (P = 0.06), I² = 64.7%							

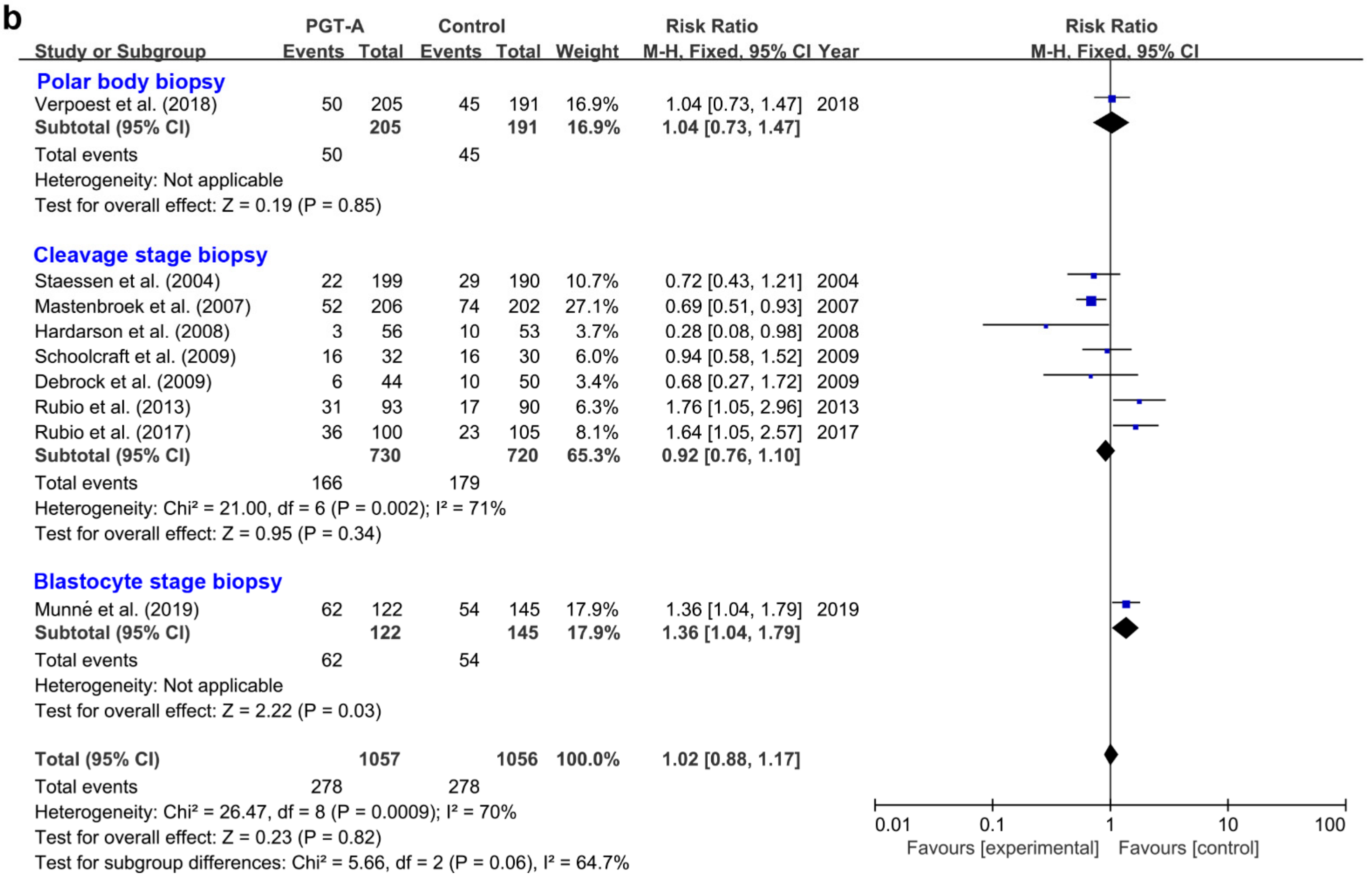

Supplemental Figure S3. The effect of PGT-A on clinical pregnancy rate. (**a**) PGT-A with different techniques of genetic testing. (**b**) PGT-A with different stages of embryo biopsy.

a

Study or Subgroup	PGT-A Events	PGT-A Total	Control Events	Control Total	Weight	Risk Ratio M-H, Random, 95% CI	Year
**FISH**							
Staessen et al. (2004)	22	199	30	190	9.5%	0.70 [0.42, 1.17]	2004
Mastenbroek et al. (2007)	61	206	88	202	14.5%	0.68 [0.52, 0.88]	2007
Hardarson et al. (2008)	5	56	13	53	4.4%	0.36 [0.14, 0.95]	2008
Debrock et al. (2009)	8	44	13	50	5.9%	0.70 [0.32, 1.53]	2009
Schoolcraft et al. (2009)	21	32	23	30	13.3%	0.86 [0.62, 1.18]	2009
Rubio et al. (2013)	36	93	18	90	10.0%	1.94 [1.19, 3.15]	2013
**Subtotal (95% CI)**		**630**		**615**	**57.6%**	**0.82 [0.57, 1.17]**	
Total events	153		185				
Heterogeneity: Tau² = 0.13; Chi² = 17.28, df = 5 (P = 0.004); I² = 71%							
Test for overall effect: Z = 1.10 (P = 0.27)							
**Comprehensive chromosomal screening**							
Rubio et al. (2017)	37	100	41	105	12.7%	0.95 [0.67, 1.34]	2017
Verpoest et al. (2018)	64	205	72	191	14.3%	0.83 [0.63, 1.09]	2018
Munné et al. (2019)	73	122	70	145	15.3%	1.24 [0.99, 1.55]	2019
**Subtotal (95% CI)**		**427**		**441**	**42.4%**	**1.00 [0.77, 1.31]**	
Total events	174		183				
Heterogeneity: Tau² = 0.03; Chi² = 5.47, df = 2 (P = 0.06); I² = 63%							
Test for overall effect: Z = 0.03 (P = 0.98)							
**Total (95% CI)**		**1057**		**1056**	**100.0%**	**0.90 [0.71, 1.13]**	
Total events	327		368				
Heterogeneity: Tau² = 0.08; Chi² = 27.10, df = 8 (P = 0.0007); I² = 70%							
Test for overall effect: Z = 0.94 (P = 0.35)							
Test for subgroup differences: Chi² = 0.80, df = 1 (P = 0.37), I² = 0%							

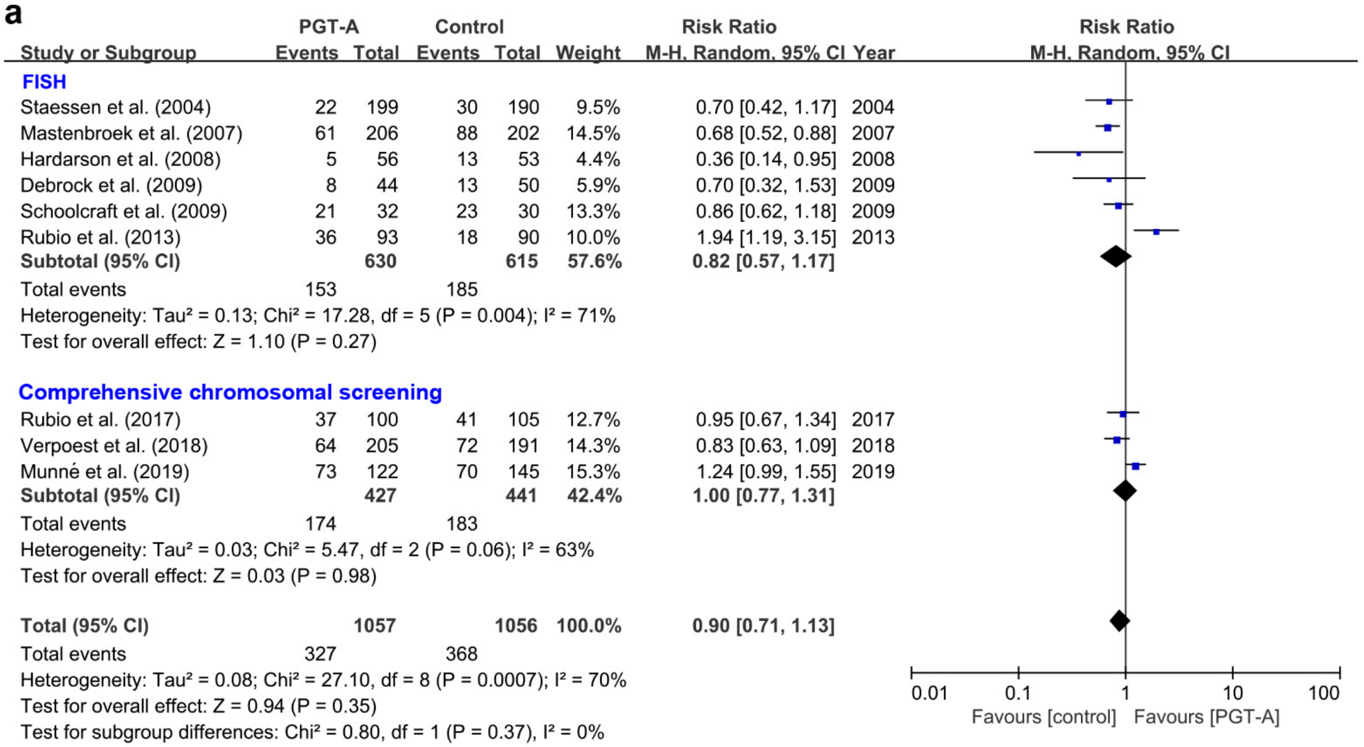

b

Study or Subgroup	PGT-A Events	PGT-A Total	Control Events	Control Total	Weight	Risk Ratio M-H, Random, 95% CI	Year
**Polar body biopsy**							
Verpoest et al. (2018)	64	205	72	191	14.3%	0.83 [0.63, 1.09]	2018
**Subtotal (95% CI)**		**205**		**191**	**14.3%**	**0.83 [0.63, 1.09]**	
Total events	64		72				
Heterogeneity: Not applicable							
Test for overall effect: Z = 1.35 (P = 0.18)							
**Cleavage stage biopsy**							
Staessen et al. (2004)	22	199	30	190	9.5%	0.70 [0.42, 1.17]	2004
Mastenbroek et al. (2007)	61	206	88	202	14.5%	0.68 [0.52, 0.88]	2007
Hardarson et al. (2008)	5	56	13	53	4.4%	0.36 [0.14, 0.95]	2008
Schoolcraft et al. (2009)	21	32	23	30	13.3%	0.86 [0.62, 1.18]	2009
Debrock et al. (2009)	8	44	13	50	5.9%	0.70 [0.32, 1.53]	2009
Rubio et al. (2013)	36	93	18	90	10.0%	1.94 [1.19, 3.15]	2013
Rubio et al. (2017)	37	100	41	105	12.7%	0.95 [0.67, 1.34]	2017
**Subtotal (95% CI)**		**730**		**720**	**70.3%**	**0.84 [0.63, 1.13]**	
Total events	190		226				
Heterogeneity: Tau² = 0.09; Chi² = 17.90, df = 6 (P = 0.006); I² = 66%							
Test for overall effect: Z = 1.14 (P = 0.26)							
**Blastocyte stage biopsy**							
Munné et al. (2019)	73	122	70	145	15.3%	1.24 [0.99, 1.55]	2019
**Subtotal (95% CI)**		**122**		**145**	**15.3%**	**1.24 [0.99, 1.55]**	
Total events	73		70				
Heterogeneity: Not applicable							
Test for overall effect: Z = 1.89 (P = 0.06)							
**Total (95% CI)**		**1057**		**1056**	**100.0%**	**0.90 [0.71, 1.13]**	
Total events	327		368				
Heterogeneity: Tau² = 0.08; Chi² = 27.10, df = 8 (P = 0.0007); I² = 70%							
Test for overall effect: Z = 0.94 (P = 0.35)							
Test for subgroup differences: Chi² = 6.69, df = 2 (P = 0.04), I² = 70.1%							

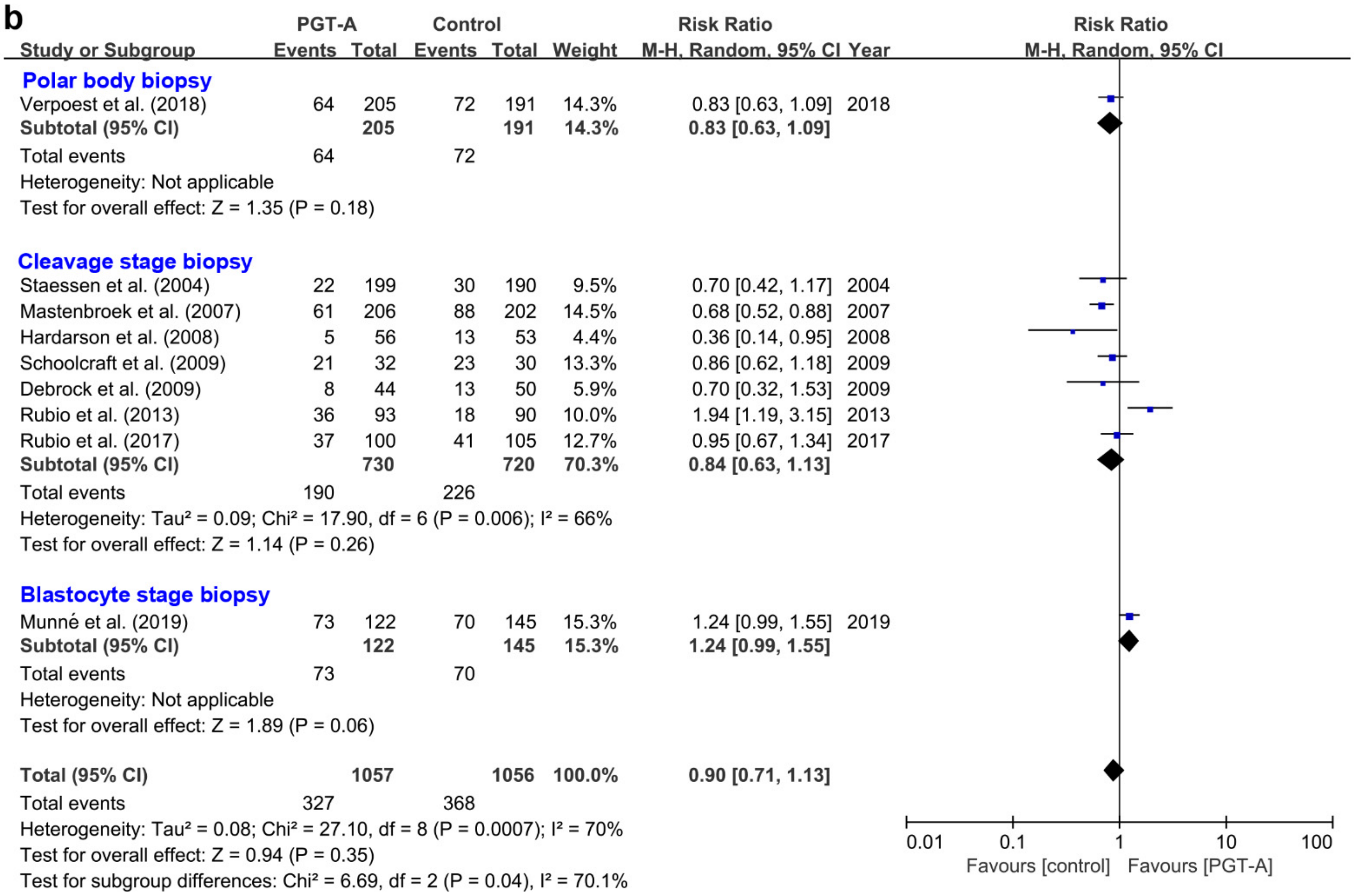

Supplemental Figure S4. The effect of PGT-A on miscarriage rate. (**a**) PGT-A with different techniques of genetic testing. (**b**) PGT-A with different stages of embryo biopsy.

a

Study or Subgroup	PGT-A Events	PGT-A Total	Control Events	Control Total	Weight	Risk Ratio M-H, Random, 95% CI	Year
**FISH**							
Staessen et al. (2004)	7	199	10	190	10.5%	0.67 [0.26, 1.72]	2004
Mastenbroek et al. (2007)	37	206	36	202	24.4%	1.01 [0.66, 1.53]	2007
Hardarson et al. (2008)	7	56	6	53	9.4%	1.10 [0.40, 3.07]	2008
Debrock et al. (2009)	2	44	5	50	4.6%	0.45 [0.09, 2.23]	2009
Schoolcraft et al. (2009)	5	32	7	30	9.2%	0.67 [0.24, 1.88]	2009
Rubio et al. (2013)	6	93	4	90	7.0%	1.45 [0.42, 4.97]	2013
**Subtotal (95% CI)**		**630**		**615**	**65.1%**	**0.93 [0.67, 1.27]**	
Total events	64		68				
Heterogeneity: Tau² = 0.00; Chi² = 2.39, df = 5 (P = 0.79); I² = 0%							
Test for overall effect: Z = 0.47 (P = 0.64)							
**Comprehensive chromosomal screening**							
Rubio et al. (2017)	1	100	16	105	3.0%	0.07 [0.01, 0.49]	2017
Verpoest et al. (2018)	14	205	27	191	17.7%	0.48 [0.26, 0.89]	2018
Munné et al. (2019)	10	122	16	145	14.2%	0.74 [0.35, 1.58]	2019
**Subtotal (95% CI)**		**427**		**441**	**34.9%**	**0.43 [0.18, 1.05]**	
Total events	25		59				
Heterogeneity: Tau² = 0.36; Chi² = 5.47, df = 2 (P = 0.07); I² = 63%							
Test for overall effect: Z = 1.86 (P = 0.06)							
**Total (95% CI)**		**1057**		**1056**	**100.0%**	**0.72 [0.50, 1.03]**	
Total events	89		127				
Heterogeneity: Tau² = 0.10; Chi² = 12.19, df = 8 (P = 0.14); I² = 34%							
Test for overall effect: Z = 1.78 (P = 0.07)							
Test for subgroup differences: Chi² = 2.54, df = 1 (P = 0.11), I² = 60.7%							

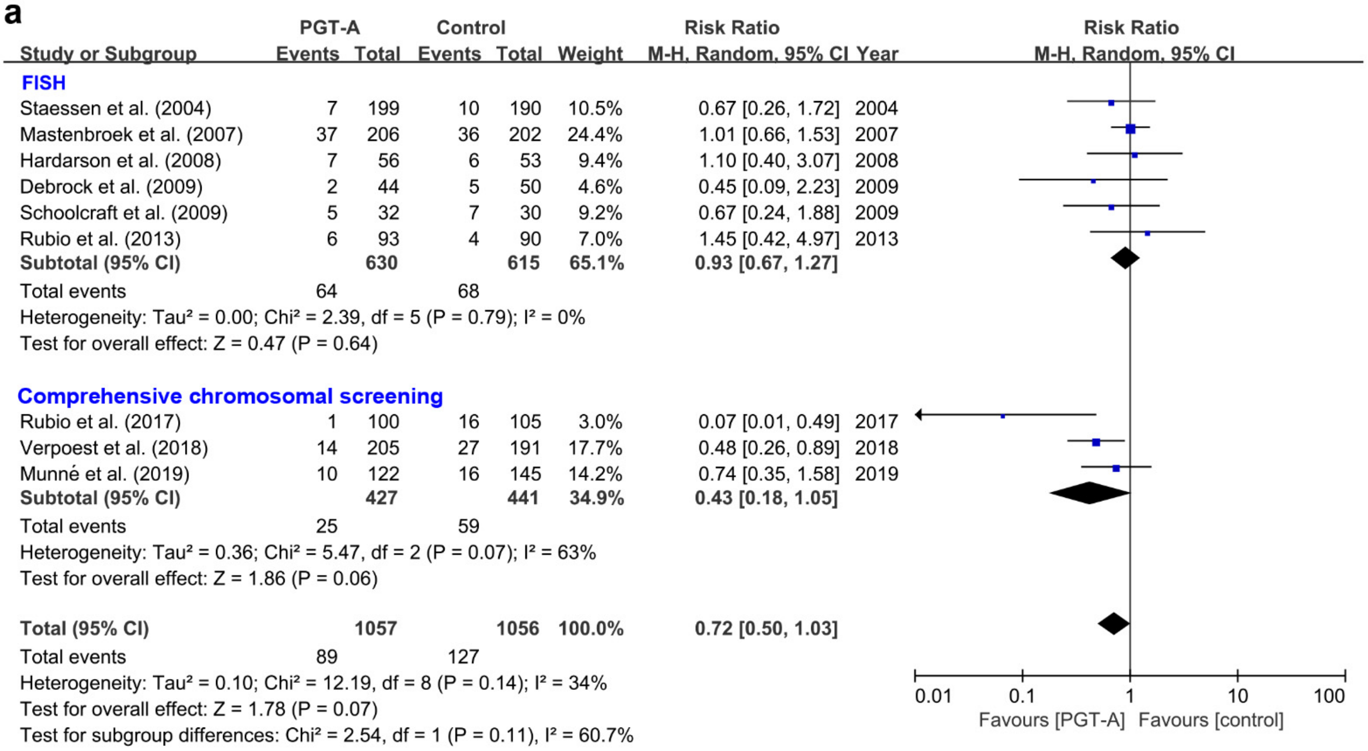

b

Study or Subgroup	PGT-A Events	PGT-A Total	Control Events	Control Total	Weight	Risk Ratio M-H, Random, 95% CI	Year
**Polar body biopsy**							
Verpoest et al. (2018)	14	205	27	191	17.7%	0.48 [0.26, 0.89]	2018
**Subtotal (95% CI)**		**205**		**191**	**17.7%**	**0.48 [0.26, 0.89]**	
Total events	14		27				
Heterogeneity: Not applicable							
Test for overall effect: Z = 2.32 (P = 0.02)							
**Cleavage stage biopsy**							
Staessen et al. (2004)	7	199	10	190	10.5%	0.67 [0.26, 1.72]	2004
Mastenbroek et al. (2007)	37	206	36	202	24.4%	1.01 [0.66, 1.53]	2007
Hardarson et al. (2008)	7	56	6	53	9.4%	1.10 [0.40, 3.07]	2008
Schoolcraft et al. (2009)	5	32	7	30	9.2%	0.67 [0.24, 1.88]	2009
Debrock et al. (2009)	2	44	5	50	4.6%	0.45 [0.09, 2.23]	2009
Rubio et al. (2013)	6	93	4	90	7.0%	1.45 [0.42, 4.97]	2013
Rubio et al. (2017)	1	100	16	105	3.0%	0.07 [0.01, 0.49]	2017
**Subtotal (95% CI)**		**730**		**720**	**68.1%**	**0.77 [0.48, 1.25]**	
Total events	65		84				
Heterogeneity: Tau² = 0.15; Chi² = 9.61, df = 6 (P = 0.14); I² = 38%							
Test for overall effect: Z = 1.06 (P = 0.29)							
**Blastocyte stage biopsy**							
Munné et al. (2019)	10	122	16	145	14.2%	0.74 [0.35, 1.58]	2019
**Subtotal (95% CI)**		**122**		**145**	**14.2%**	**0.74 [0.35, 1.58]**	
Total events	10		16				
Heterogeneity: Not applicable							
Test for overall effect: Z = 0.77 (P = 0.44)							
**Total (95% CI)**		**1057**		**1056**	**100.0%**	**0.72 [0.50, 1.03]**	
Total events	89		127				
Heterogeneity: Tau² = 0.10; Chi² = 12.19, df = 8 (P = 0.14); I² = 34%							
Test for overall effect: Z = 1.78 (P = 0.07)							
Test for subgroup differences: Chi² = 1.48, df = 2 (P = 0.48), I² = 0%							

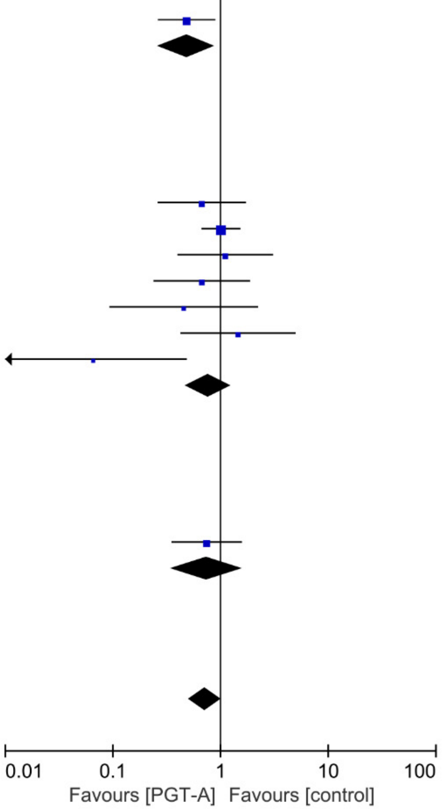

Supplemental Figure S5. The effect of PGT-A on multiple pregnancy rate. (**a**) PGT-A with different techniques of genetic testing. (**b**) PGT-A with different stages of embryo biopsy.

a

Study or Subgroup	PGT-A Events	PGT-A Total	Control Events	Control Total	Weight	Odds Ratio M-H, Random, 95% CI	Year
**FISH**							
Staessen et al. (2004)	4	199	6	190	14.7%	0.63 [0.17, 2.26]	2004
Mastenbroek et al. (2007)	10	206	14	202	28.7%	0.69 [0.30, 1.58]	2007
Hardarson et al. (2008)	0	56	2	53	3.0%	0.18 [0.01, 3.89]	2008
Debrock et al. (2009)	1	44	1	50	3.5%	1.14 [0.07, 18.77]	2009
**Subtotal (95% CI)**		**505**		**495**	**49.8%**	**0.65 [0.33, 1.26]**	
Total events	15		23				
Heterogeneity: Tau² = 0.00; Chi² = 0.84, df = 3 (P = 0.84); I² = 0%							
Test for overall effect: Z = 1.29 (P = 0.20)							
**Comprehensive chromosomal screening**							
Rubio et al. (2017)	8	100	3	105	13.4%	2.96 [0.76, 11.48]	2017
Verpoest et al. (2018)	14	205	24	191	36.8%	0.51 [0.26, 1.02]	2018
**Subtotal (95% CI)**		**305**		**296**	**50.2%**	**1.11 [0.20, 6.16]**	
Total events	22		27				
Heterogeneity: Tau² = 1.25; Chi² = 5.14, df = 1 (P = 0.02); I² = 81%							
Test for overall effect: Z = 0.12 (P = 0.90)							
**Total (95% CI)**		**810**		**791**	**100.0%**	**0.72 [0.42, 1.23]**	
Total events	37		50				
Heterogeneity: Tau² = 0.08; Chi² = 6.03, df = 5 (P = 0.30); I² = 17%							
Test for overall effect: Z = 1.19 (P = 0.23)							
Test for subgroup differences: Chi² = 0.33, df = 1 (P = 0.57), I² = 0%							

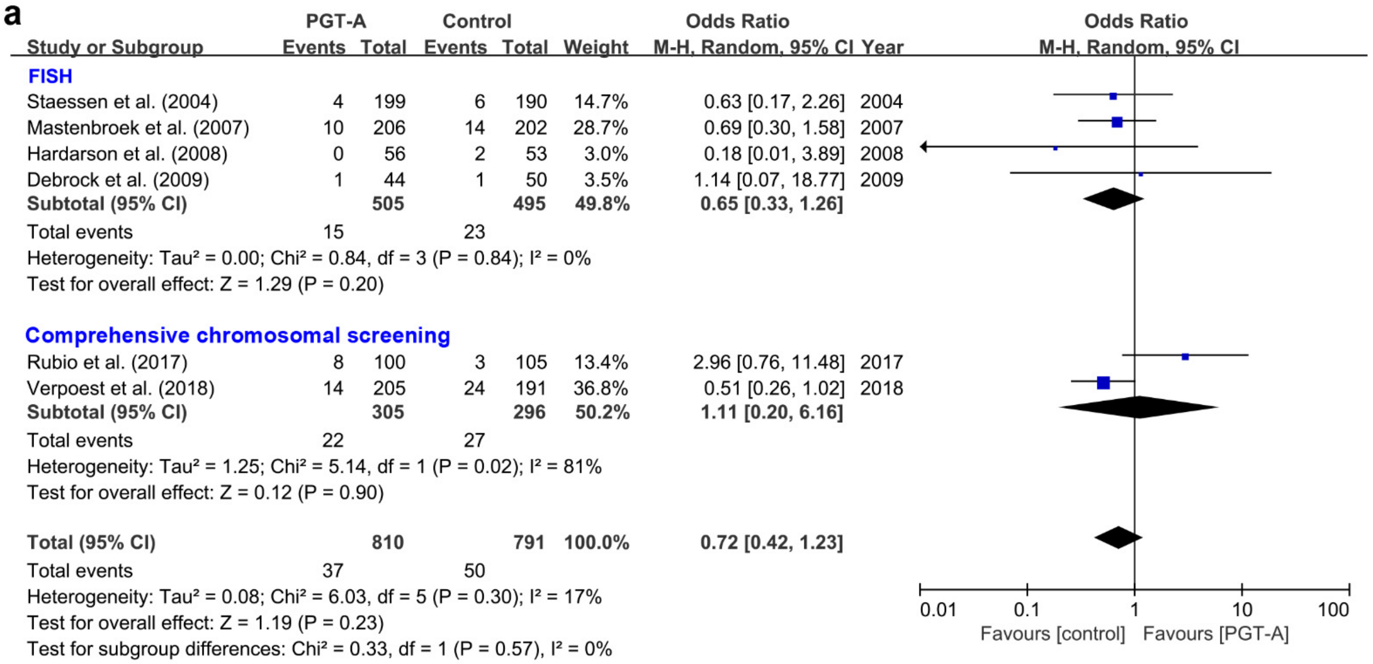

b

Study or Subgroup	PGT-A Events	PGT-A Total	Control Events	Control Total	Weight	Odds Ratio M-H, Random, 95% CI	Year
**Polar body biopsy**							
Verpoest et al. (2018)	14	205	24	191	36.8%	0.51 [0.26, 1.02]	2018
**Subtotal (95% CI)**		**205**		**191**	**36.8%**	**0.51 [0.26, 1.02]**	
Total events	14		24				
Heterogeneity: Not applicable							
Test for overall effect: Z = 1.91 (P = 0.06)							
**Cleavage stage biopsy**							
Staessen et al. (2004)	4	199	6	190	14.7%	0.63 [0.17, 2.26]	2004
Mastenbroek et al. (2007)	10	206	14	202	28.7%	0.69 [0.30, 1.58]	2007
Hardarson et al. (2008)	0	56	2	53	3.0%	0.18 [0.01, 3.89]	2008
Debrock et al. (2009)	1	44	1	50	3.5%	1.14 [0.07, 18.77]	2009
Rubio et al. (2017)	8	100	3	105	13.4%	2.96 [0.76, 11.48]	2017
**Subtotal (95% CI)**		**605**		**600**	**63.2%**	**0.89 [0.44, 1.78]**	
Total events	23		26				
Heterogeneity: Tau² = 0.10; Chi² = 4.72, df = 4 (P = 0.32); I² = 15%							
Test for overall effect: Z = 0.34 (P = 0.74)							
**Total (95% CI)**		**810**		**791**	**100.0%**	**0.72 [0.42, 1.23]**	
Total events	37		50				
Heterogeneity: Tau² = 0.08; Chi² = 6.03, df = 5 (P = 0.30); I² = 17%							
Test for overall effect: Z = 1.19 (P = 0.23)							
Test for subgroup differences: Chi² = 1.23, df = 1 (P = 0.27), I² = 18.8%							

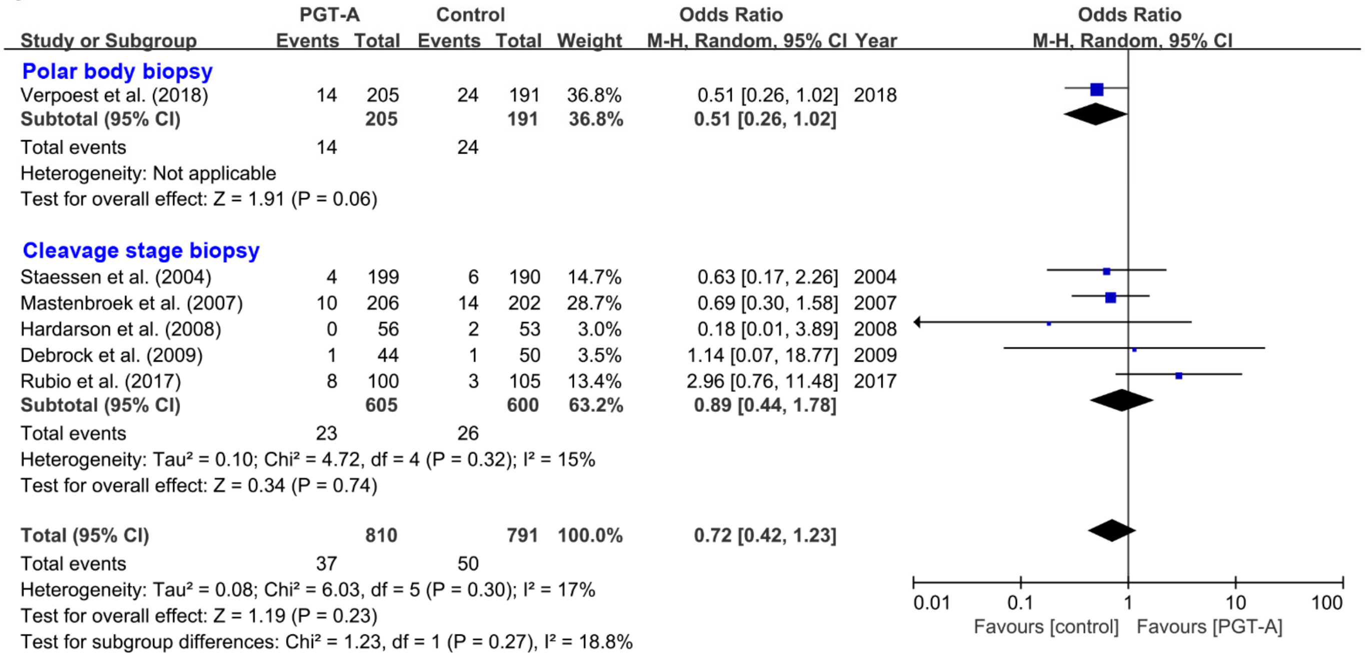

Supplement: Supplementary file 1 [file jcm-10-03895-s001.zip › jcm-1284683-supplementary.pdf]
